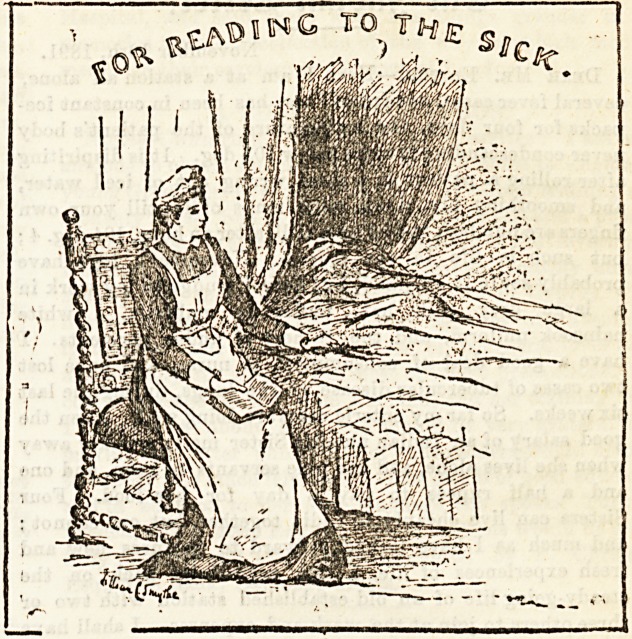# The Hospital Nursing Supplement

**Published:** 1891-12-19

**Authors:** 


"The Hospital" Nursing Mirror.

Being the Extra Nursing Supplement of "The Hospital" Newspaper.

Contributions for this Supplement should be addressed to the Editor. The Hospital, 140, Strand, London, W.C., and should have the word "Nursing" plainly written in left-hand top corner of the envelope.

### En Passant.

**A CURE FOR HYSTERIA.**—A nurse, whose name we shall not give, has been had up before her Board for flicking an hysterical patient with a wet towel. The Board found the charge of ill-usage not proven, but asked the nurse not to resort to the same method of treatment again. We consider the nurse got off easily, for such violent treatment should certainly never be used except under medical direction. There are no cases which need such careful nursing as those of hysteria; there are no cases of which nurses comprehend less. Perhaps when asylum training is open to nurses as a completion of their education, we may hope that they will better understand how to attend to nervous, hysterical, and mental cases.

**GUILD OF ST. VERONICA.**—As many of the readers of The Hospital have asked for information about the Guild of St. Veronica, the following short account may be of interest to them. Its objects are three in number: I. To form a bond of union among those who are engaged in the work of nursing the sick. II. To aid the spiritual life of its members under the peculiar and special difficulties of their duties. III. To help its members to realise that nursing the sick is properly a religious work, and should be performed as a labour of love. The rules for the members are few, but strict, and are specially formed to help nurses in their spiritual life, the founder of the Guild having had practical experience of a nurse's difficulties during his connexion with hospitals. Further particulars can be obtained from the Secretary, Miss F. Robertson-Macdonald, 9, Great Bedford Street, Bath.

**A NEW HOME AT GLASGOW.**—Mr. John Wilson on December 4th, opened a new nursing home in connection with the Glasgow Western Infirmary. By the additional buildings forty-seven new bedrooms have been provided, making in all eighty-seven separate bedrooms for the nursing staff. The building, which is oblong, comprises three flats, and on the ground floor are situated the Sisters' drawing-room, nurses' sitting-room, recreation and reading rooms. The drawing-room and sitting-room are richly furnished, a grand pianoforte being in the former and a cottage piano in the sitting-room. All the rooms are large and airy. Mr. Wilson, in declaring the home open, said that it long been the desire of the managers to have the Nurses' Home large enough to accommodate the whole of the nursing staff on the premises, and it would be observed that the home was detached from the wards. They could easily understand how beneficial this was, both in the interests of the patients and the nurses. The home would be found replete with every necessary comfort. The public rooms and bedrooms had been furnished with a view to making the nurses feel that they were indeed at home. Professor Buchanan said that there was no wonder good accommodation was required, because the type of nurse had changed somewhat to what it used to be. Nowadays the nurses were highly talented and of great intelligence, and carried out the doctor's wishes to the utmost. They could not expect that nurses of that calibre and upbringing could be accommodated with what used in the old days to be considered sufficient. When the patients enjoyed these benefits it was only right that the nurses should have them. Professor M'Call Anderson, Dr. Paterson, Mr. M'Ewen, and others, also spoke, and then the company were provided with tea.

**TENBY TITTLE-TATTLE.**—"This Nursing Institute at Tenby is a very cliquey affair," said Dr. Lock to the Pembroke Board of Guardians at their late meeting; and the Board agreed with Dr. Lock, who is one of their Medical Officers, and decided that he was not forced to have pauper patients attended by the district nurse. Then Dr. Lock complained that the nurse had been two days in the town before he heard anything about her, and that she prescribed for the patients. All this savours very much of provincial jealousy and petty strife; but there is one serious point—did the nurse really prescribe for the patients? This is an accusation so serious as to need the immediate attention of the Institute, and an authoritative and conclusive answer. Certainly someone has been wanting in tact, even if nothing worse has happened.

**SHORT ITEMS.**—Miss Josephine Wake, to whom the success of the Brighton District Nursing Society is largely due, is going abroad for her health.—The gross takings at the Preston Doll Show were £105; the nurse dolls were much admired.—The Secretary, the Midwives' Institute, 12, Buckingham Street, Strand, would be glad to send blanks to midwives or monthly nurses who will fill them in with answers of mothers to the question: "In how many of your confinements have you been attended by a doctor, his assistant, a medical student, a midwife, or a neighbour; and what did you pay?"—Sister Harriet Rose has received a grant from the S.P.C.K., towards the Cottage Hospital at Mortate, to which she is shortly going out.—Dr. Little constantly employs a blind Masseuse.—We hope to give as usual this year brief accounts of how Christmas was kept in the hospitals all over the kingdom. Will Matrons and nurses please send us these accounts as early as possible, written briefly, and on one side of the paper only?

**GLASGOW SICK POOR NURSING ASSOCIATION.**—A systematic course of lectures on Nursing is delivered during the winter, to the nurses of the above Association, and to which all ladies are invited. The course began on Tuesday evening, the 10th of November, at eight p.m., and will be continued every Tuesday evening at the same hour. The following is a list of the lectures to be given after Christmas:—January 5th—Dr. R. Cowan Lees, "The Pulse. The Respiration. External Treatment of Fever." January 12th—Dr. R. H. Parry, "Position and Rest in some Surgical Affections." January 19th—Dr. R. H. Parry, "Wounds and some of the causes which prevent them healing." January 26th—Dr. R. H. Parry, "Operations and Shock." February 2nd—Dr. H. St. Clair Gray, "General Health. Rules for Patients prior to Accouchement. Rules for Monthly Nurses. Antiseptics — their meaning and application to Monthly Nursing." February 9th—Dr. H. St. Clair Gray, "Choice and preparation of a Lying-in room. Diet of Patient. Care of Patient during Convalescence." February 16th.—Dr. H. St. Clair Gray, "The Newly-born Child. Diseases of the Newly-born and their Treatment. Artificial Feeding of Infants." Extra lectures if necessary: Dr. Caldwell Smith, "Hygiene of Infancy and School Life." Dr. R. Cowan Lees, "Foods and Drinks. The necessity for a mixed Diet. Digestion." Dr. R. H. Parry, "Symptoms on Cerebral and Spinal Diseases. How to observe and report them." Dr. St. Clair Gray, "The Incubator—methods of raising Premature and Sickly Infants. The Choice of a Wet Nurse." Both the nurses and ladies of Glasgow may consider themselves privileged to be able to attend such a complete course of lectures.

### Lectures on Surgical Ward Work and Nursing.

By Alexander Miles, M.D. (Edin.), F.R.C.S.E.

Lecture XXXIX.—NEEDLES AND TREPHINING INSTRUMENTS.

Needles are employed to sew up wounds, whether made by accident, or by the knife in the course of an operation. Some surgeons prefer a straight needle, which differs from an ordinary sewing needle, in having the blade somewhat flattened

Fig. 1.

from side to side, and the eye considerably larger. Many use the half-curved needle, while perhaps the most frequently employed is the curved needle, on account of the greater ease and rapidity with which sutures may be inserted by it (fig. 1).

Fig. 2.

Fig. 3.

Needle holders are, as a rule, dispensed with by ordinary surgeons, but by some they are found to be of advantage, and many patterns have been introduced, characterised, however, more by the ingenuity of their mechanism than by their practical utility. Some are made to be used with any form of needle, *e.g.* (fig. 2), which represents a pair of dressing forceps, which may also be used as a needle holder, being adapted with a longitudinal groove into which the needle fits. Figures 3 and 4 represent two forms of needle holder used by ophthalmic surgeons. The Hagedorn needle holder can only be used with the flat-bladed needles employed by that surgeon.

Instruments used in Trephining the Skull.—The operation known as trephining or trepanning the skull con-

Fig. 6.

Fig. 4.

Fig. 8.

sists in the removal of a circle of bone from the vault, in order to obtain access to the contents of the cranium, or to facilitate the elevation of depressed fragments when the skull has been fractured. The instruments used in the successive steps of the operation are these: (1) A razor of the ordinary kind, with which to shave the region of the head to

Fig. 5.

be operated upon; (2) a scalpel, or small bistoury, to make the incision through the tissues overlying the bone; (3) A periosteum separator, with which to raise the pericranium, as the covering of the skull bones is called (fig. 5); (4) blunt hooks to retract the edges of the scalp wound; (5) dissecting forceps (fig. 6) may be found useful in this operation, as,

Fig. 7.

Fig. 7a.

indeed, they are in almost every ordinary surgical operation; (6) artery forceps (Wells' or Pean's) will, of course, be needed to secure the blood vessels cut in making the scalp incision. (7) The Trephine.—This is the instrument used to saw out the bone circle, and from it the operation gets its name. It consists of a small round saw, varying in diameter from ½ to one inch, mounted on a strong hollow metal shaft, about 3½ or 4 inches long, to which is attached at right angles, a thick, strong handle. Running down through the hollow of the shaft is a movable centre-pin, which may be made to project beyond the level of the saw teeth, or be entirely withdrawn, as required. It is fixed in the desired position by a screw on the side of the shaft. In some instruments only the free edge of the blade is serrated, *e.g.* (fig. 7), and in others the serrations are carried along the outer aspect of the blade (fig. 7A). In using trephine, the centre pin is first projected from 1-16th to 1-8th of an inch beyond the level of the saw, and there fixed firmly. The point of it is then fixed into the centre of the area of bone to be removed, and in this way the saw is steadied until it has made a groove for itself in the bone. So soon as this has been accomplished the centre pin is completely withdrawn, lest it should interfere with the onward progress of the saw, or, perforating the bone before the saw, injure the membranes of the brain underneath. The saw is then gradually worked through the bone with a rotatory motion, as in using a bradawl, great care being necessary on account of the fact that the skull is not of the same thickness all over, and there is consequently a danger of one part of the circle being through early, and of the saw opposite that part damaging the membranes underneath: To prevent such an accident the trephine must be frequently removed and

FIG. 9.

FIG. 10.

FIG. 11.

the groove measured all round. This is most conveniently done by an ordinary tooth-pick, blunted at the point, which also serves to remove the bone dust from the groove. (8) The bone being sawn through all round, the circle has now to be lifted out, and for this purpose the Trephine forceps are employed. These are made on the same principle as dissecting forceps, but the blades are rounded so as to adapt themselves to a curved object (fig. 8). (9) An instrument called the Lenticular is sometimes used at this step of the operation to plane the edges of the hole in the bone. It has a stem with sharp edges which scrape off any projections, and these fall into a small basin-shaped button at the foot. It is not often used (fig. 9). (10) When the operation is performed for fracture of the skull, it is often necessary to saw off projecting ledges of bone, and this is done by means of Hey's saw (fig. 10). This is really two small saws of different shapes fixed on to a long, strong handle. (11) Another instrument also used in cases of fracture is the Elevator (fig. 11). As its name implies, it is used to raise up any depressed fragments of bone which are pressing on the subjacent brain. It is to be distinguished from the periosteum separator, being roughened on one aspect to prevent it slipping from under the bone. (12) The other instruments used in the operation will depend on the object for which it is being performed—*e.g.*, the removal of a tumour or foreign body, the evacuating of an abscess, or the elevation of depressed bone. In all conditions as asepsis is the key of success, the greatest care must be taken in the purification of instruments, hands, dressings, &c.

The illustrations are used by kind permission of Messrs. J. Weiss and Sons.

### THE FADING YEAR.

THE year is fading, and we look regretfully on the withered leaves at our feet which hung so lately in rich profusion and varied hues upon the trees. Their glory has departed never to return. It is well at times to remember that our life too is short. But a few years ago we were young and bright and strong; with hope high within us, we imagined all sorts of possibilities for the future. Now our castles in the air have crumbled into dust, our great aims have been prevented in their fulfilment, and everything seems slipping away from us; first our youth, then our health and strength. Mayhap even our lives will soon be gone, and we shall have to confess that we have left undone those things which we ought to have done, happy if we need not add that we have done those things which we ought not. Oh, blasted hopes! oh, lost opportunities! the waning year warns us to gather up the fragments of time and opportunities so that everything be not lost.

But though we bring so little to perfection, though the very best among us, those who have the highest aims, and those who work hardest in their callings, feel their hearts sinking as they realise how unsatisfactory are all their efforts; yet these very facts point to another life beyond the grave, "The life that hath no ending, the deathless life is there." In that blessed life all our shortcomings will be amended, all our tangled threads smoothed out. Christ has given us this blessed hope in various forms. He tells us that in His Father's house there are many mansions prepared for them that love Him. St. Paul reminds us that Christians would be of all men the most miserable if this world were all we had to look to, and in his epistle, I. Cor. chap. xv., he gives a sure hope to cling to, namely, that these poor bodies so sick, so weak, so suffering now, shall rise from the dust as glorious bodies to be ever with the Lord. Not the rich, the wise, the mighty, the successful in this life are those who shall be taken into glory; but the meek, the poor in spirit, they who have been voted failures by their fellow creatures, and who feel they are unprofitable servants, it is they who are meet for the Kingdom of Heaven.

The withered leaves, says the Christian poet, "fall, forgotten to abide, 'tis all their portion and they ask no more," while "Man's portion is to die and rise again—yet he complains." We must work on then in hope, steadfast, unmoveable, forasmuch as we know that our labour is not in vain in the Lord.

### Our Indian Letter.

November 10th, 1891.

Dear Mr. Editor,—Here I am at a station all alone, several fever cases, one enteric case has been in constant ice-packs for four days, the temperature of the patient's body never condescending to come below 103 deg. It is dispiriting after rolling a patient in a sheet wrung out of iced water, and smoothing him down with lumps of ice till your own fingers are numbed, to find he is still, after an hour, 104 deg. 4; but such is life in the tropics. November's fogs have probably set in in England, but here I trudge to my work in a large grey pith hat, under an umbrella, a white nainsook uniform, and the thinest of under garments. I have a good deal of consumption to nurse, and have lost two cases of tubercular disease of the lungs, during the last six weeks. So far my enteric cases are doing well. Even the good salary of an Indian nursing Sister melts quickly away when she lives alone, and has nine servants to keep, and one and a half rupees to pay a day for her food. Four Sisters can live cheaply in India together, but one cannot; and much as I have looked forward to pastures new and fresh experiences of life, I look regretfully back on the steady-going life of an old-established station with two or three others to join at the work and expenses. I shall have a warm and not altogether disinterested welcome for the Sisters who will join me shortly from England.

We had a sad case here a fortnight ago. A corporal fell down some steps and fractured his skull. He was brought into hospital, and died in five days; at 11 p.m. the next day he was buried, and a few hours afterwards his wife arrived from England to join him. Had the authorities known, the funeral might have been delayed, and she would have had the melancholy pleasure of at least seeing his face. They are prompt in the army—dead one day, buried as quickly as possible the next. This week, the widow was told a passage had been taken for her on H.M.S. Serapis, and she had to leave for England that day; a few hours' notice to settle her affairs and take a last farewell look at the grave. She came to me in her sorrow; I helped her as I could, and suggested her taking up nursing. She is young and strong and pleasant looking, her four children are dead, and she has acted as maid to at least two ladies, if not more, who would recommend her. She will be home about December 4th or 6th. Would any Lady Superintendent of private nurses, reading this, kindly send their forms of application to Mrs. K., The Hospital Office, 140, Strand, W.C.? I would like particularly to recommend her to Miss Wilson, for the Workhouse Nursing Association. I would write, but, unfortunately, I have not her address. This woman has been twice in India, so is used to travelling.

Lately, the newspapers have been teeming here with a battle between "the Non-Combatant" and "the Combatant Officers" on the subject of the new medical titles. It has created a good deal of ill-feeling out here, and for weeks there have been a succession of abusive letters on both sides. I enclose two poems from the "Pioneer" on the subject. One combatant officer styling himself "Captain," wrote a very puerile ungentlemanly letter, and stated that he felt up till now that his title at least had given him an attraction in the eyes of the fair sex, but now he had to share it with vets. and doctors; he even ventured to raise a sneer against the lady nurses, which sneer we have all been too dignified to notice.

The most amusing thing is that these "combatant officers," as they have styled themselves, do not hesitate to interfere, and vaunt their knowledge in medical matters. One Major came into a ward one day, "Any lancers seriously ill, Sister?" I remarked that one was in great danger. Hæmorrhage had set in profusely, and he had pneumonia as well. He asked to look at the chart, pointed to it with a superior air to a brother officer, "Nothing much wrong here; temperature down." Another officer comes round, feels pulses, studies the charts and examines the tongues, and criticises the treatment. He even occasionally gives a little instruction. He told me one day that the tongue was the "index of the stomach," and further asked if I knew it. This morning two officers came into the ward, the doctor going round. They never apologised, or asked leave. The Captain felt the patient's pulse; remarked to me, "This patient's pulse is very slow." I remarked quietly, "Do you think so? it was 105 a few minutes ago." "Ah, yes," he answered, "my pulse is just about the same." He then asked what was the matter with the man. I told him enteric fever. He then asked how long he had been ill. I said thirty days. He said, "Then it can't be enteric fever; that only lasts twenty-one days; and I have always heard that if a man has it over twenty-one days he is bound to die." I told him I had nursed a great many who had been ill longer than twenty-one days, and had lived. I have a case in the ward now, who had three relapses before I came; is not well yet, and I have been here six weeks, and this was a case where the man's temperature was over 107, and I don't think he is at all likely to die. Indian ideas of nursing are amusing. One lady asked me if I sat by the patient's bed all day.

An apothecary here performs the functions of a house surgeon, a ward sister, and a dispenser. He also exercises discipline in the wards; they felt our coming very much. An apothecary in a sister's ward sinks entirely into the dispenser. His military rank, with a few exceptions of honorary apothecary captains and honorary apothecary lieutenants, is that of warrant officer, that is equivalent to a bandmaster, sergeant-major, and schoolmaster in the Army; they are chiefly Eurasians; there are very few Europeans among them. They do great a deal of the medical work, are much looked down upon, and are badly paid. There are splendid men here, and they work like Trojans; but my past experience of apothecaries has been the reverse. I found them most difficult to work with, and not enthusiastic in their work.

The military orderly out here is a mistake; he is a voluntary worker. In some few cases they receive slight pay; they do this, which is lighter work, instead of regular duty, and are, as a rule, most unsatisfactory.

Though comrades, the patients don't care to be nursed by them as a rule. They are not properly trained, though a few hold certificates of training. After attending an ambulance class, they go through an examination—a very elementary one—they are nearly always passed and are given an ambulance certificate. One, whom I know, that was passed, declared in his examination that a man possessed two livers. In India most people find one rather too much; I don't think anyone wants two out here except for a change. In the old days the orderlies gave medicines and stimulants, or drank the latter themselves, as they thought fit; there was very little surpervision over them. Just before I came to this station the Padre asked one of the orderlies how he liked the idea of Sisters coming; he promptly said, "Not at all, we had hoped to form the knuckles of an 'Orspital Corpse, but these Sisters coming has put us all out of joint."

N.B.—This story is vouched for by the principal Padre.

There is a really authentic case of a nurse in the Bengal Presidency whose temperature this summer rose nightly to 119°. Five doctors sat round the bed to see that she played fair. It is said that the room thermometer was used, as a clinical one could not register it. She lives to tell the tale, and is not much the worse. How was it done? The doctors believe in the temperature.

An Army Hospital Corps is much needed here, formed of Europeans we have only, except the irregular help of soldier orderlies, the Native Hospital Corps to assist us. Cholera has broken out at Lucknow and Lahore, though this is not the cholera season. At Lucknow one poor man who, last winter, was safely piloted through a long enteric case, and had returned to duty as a gunner full of health and strength, has fallen a victim to the fell disease. Mrs. Meecham, wife of Colonel Meecham, the officer commanding the Scottish Rifles, succumbed at Jabbulpore of the disease four days after leaving Lucknow. Allahabad was the scene of terrible cholera this summer. Among the troops, many recovered, but out of the South Wales Borderers alone, 32 died. Within a year at that station they have lost nearly fifty men and one officer, Major Bromhead, V.C., the remainder having died of consumption and fever. Consumption and chest diseases are very common there.

AN OLD FOGEY.

### Everybody's Opinion.

*[Correspondence on all subjects is invited, but we cannot in any way be responsible for the opinions expressed by our correspondents. No communications can be entertained if the name and address of the correspondent is not given, or unless one side of the paper only be written on.]*

### NOTICE OF APPOINTMENT.

"BRISBANE" writes: May I ask you to take notice of the uncourteous behaviour of the Secretary (or possibly the Committee) of the Rotunda Hospital, Dublin? I answered their advertisement in September for a Lady Superintendent, enclosing copies of testimonials, and up to date (December 7th) I have not heard a word from them. I have written twice to know if the post was filled u , the last time sending a stamp for a reply, and requesting that if it was not, nor likely to be soon, my name might be withdrawn and testimonials returned. Women who have to earn their living cannot afford to wait in this way; and if the applications were too numerous to be answered by post, at least a notification that the post was filled might be inserted in THE HOSPITAL.

[The Rotunda appointment is filled up. We have had no notice of the appointment either.]

### SICK NURSES.

ANNIE MERCIER, Kemerton Rectory, writes: May I inform you how gladly Sick Nurses are welcomed for a period of repose at the Home of Rest, Malvern Wells (see advertisement), where every advantage is given them at a lower fee than to the general public? The Home is in a fine position on the hill side; the house is large and well warmed, and the fare good though simple. There are a drawing-room, a library, a piano, and everything that the Committee can arrange for the comfort of visitors.

### ST. MARY'S COTTAGE HOSPITAL.

SISTER KATHERINE writes: I regret that no notice of the opening of this Cottage Hospital and Nurses' Home was sent to your periodical. Press of work is my only excuse. During the month of November, in addition to the sale of work and opening of both Homes, sixteen patients were admitted into the Cottage Hospital, over 700 visits were paid to maternity cases, and over 800 to district cases, including several severe cases of typhoid. Six nurses had cases lasting for two weeks which are not counted in the district cases. Eight pupils from St. Mary's Home were sent up to the examination of the L. O. S. in October, and all passed. The doctor sees from thirty to forty out-patients every Tuesday, and a considerable number attended daily for dressings, etc. We have had six cases of burn within the last few days. The nearest hospital is the West Ham Cottage Hospital, a mile and a half away. The Bishop of St. Albans in his speech alluded to the medical men who gave their services to the Hospital, and said there were few things grander in our memories than a recollection of the way in which medical men gave their services to the poor of London.

### A PUPIL FROM CYPRUS.

NURSE CHRISTIAN, Niconia, Cyprus, writes: I am very anxious to obtain sufficient funds to send home to England a young Greek girl in order that she may obtain training as a monthly nurse. She is a highly respectable girl and a promising pupil, having already had some teaching in the Government Hospital, Niconia. The want of a trained monthly nurse is greatly felt, there being at present not one in Cyprus. The native women now acting in that capacity are absolutely ignorant of their work, fatal cases of peurperal fever occur almost daily for want of the most ordinary precautions. The cost of passage to and from England, and her training there I estimate at about £50, and should be very grateful if by making this letter public any of your readers would help me to supply this much felt want. The girl is of good education and in addition to her own language she speaks Turkish, French, and a little English. Contributions would be gratefully received and all enquiries answered by B. Christian, Esq., The Bank, Baldock, Herts.

### "MORAL BACILLI."

"E. M." writes: Reading your comments upon an article by Mrs. Lynn-Linton made me think that what the Rev. B. Waugh has done, and is still doing, for the poor little children, has been done for many big children, who cannot feel too grateful for the chance they now have of looking forward to an independent and honourable old age, thanks to all who so kindly helped to start our R.N.P.F. We are upheld by the most gracious President in England, and nothing could so well fill the lives of lonely and sympathetic women as the care of the sick and suffering. Through the work thus provided for thousands of women, the axe is laid at the root of one source of domestic misery. I mean the marriage which, years ago, women rushed into for the sake of a home. Now that cause is removed for ever, and the full effect will be seen in the next generation, and cause the future Mrs. Lynn-Lintons to find a better subject to discuss than "Moral Bacilli."

### Appointments.

BLACKBURN WORKHOUSE INFIRMARY.—Miss Millicent Toft has been appointed Superintendent Nurse at Blackburn Workhouse Infirmary, at a salary of £40 a-year, and uniform. Miss Toft has been on the staff of the Carlisle Workhouse Infirmary, from which she has been promoted to Blackburn Infirmary. She was formerly at Withington.

INDIAN SERVICE.—Miss Beatrice Cann, of St. Mary's Hospital, has been appointed Sister in the Indian Nursing Service, and sailed on December 12th in H.M.S. "Crocodile."

### Amusements and Relaxation.

### SPECIAL NOTICE TO CORRESPONDENTS.

**Fourth Quarterly Word Competition commenced October 3rd, ends December 26th, 1891.**

Competitors can enter for *all* quarterly competitions, but no competitor can take more than *one first prize* or *two* prizes of any kind during the year.

Proper names, abbreviations, foreign words, words of less than four letters, and repetitions are barred; plurals, and past and present participles of verbs, are allowed. Nuttall's *Standard* dictionary only to be used.

N.B.—Each paper must be signed by the author with his or her real name and address. A *nom de plume* may be added if the writer does not desire to be referred to by us by his real name. In the case of all prize-winners, however, the real name and address will be published.

The word for dissection for this, the TWELFTH week of the quarter, being

"GOODWILL."

| Names. | Dec. 10th. | Totals. | Names. | Dec. 10th. | Totals. |
|---|---|---|---|---|---|
| Lightowlers | 70 | 495 | Jenny Wren | 78 | 417 |
| Bonne | 52 | 496 | Darlington | 71 | 491 |
| Morico | 80 | 566 | Nurse G. P. | — | 99 |
| Robes | — | 143 | Hetty | 69 | 407 |
| Dulcamara | 76 | 509 | Janet | — | — |
| Psyche | — | 7 | Jackanapes | — | — |
| Agamemnon | 80 | 538 | Ex Nurse | — | 99 |
| Nurse J. S. | 67 | 474 | | | |

All letters referring to this page which do not arrive at 140, Strand, London, W.C. by the *first post on Thursdays*, and are not addressed PRIZE EDITOR, will in future be disqualified and disregarded.

### The Lay of a Combatant.

(*See our "Indian Letter," page* lxx.)

He was very much a soldier
And his uniform was blue,
With tons of lace upon it
And buttons not a few.
His sword was burnished brightly
And spurs he wore of course,
You might take him for a "Gunner"
When not upon his horse.

I wrote to him a little note,
Just some hasty lines to say,
"Dear Doctor, come and look me up
I've had fever all the day."
He wrote across my letter
In the goriest of inks,
"I'll beg you to address me
As Lieutenant-Colonel Jinks."

"How many did you kill," wrote he,
"In your last little fight?
Now mind you don't exaggerate,
Give me the total right."
Full truthfully I answered him
(I'm full of truth you see)
"When last I stemmed the battle's tide
My bag was only three."

Full scornfully he smiled on me,
"So you think that you can kill.
For my sword I've but a lancet,
For my bullet but a pill.
Yet you're distanced in the running
You're not in it in the race,
Why I've stocked a little graveyard
In this very healthy place."

### The Lay of a Non-Combatant.

So now we're taunted every day
By popinjay or paltry scribe
With our presumption! Yes, they say,
With pointless jest and senseless jibe,
That Doctors, whose mere work's to save,
Are all unfit, of course, to bear
Titles that erst proclaimed the brave
And, eke says one, allured the fair.

So be it. Let warriors bold who fight
Attract the fair! We only ask—
Nay, we demand it as a right—
Room to fulfil our nobler task.
But if by titles you acclaim
Duty 'mid danger bravely done,
A record spotless, free from shame,
Then in our claims we yield to none.

Our calling grand no craven breeds,
And every hour, unknown to fame,
Are quietly performed such deeds
As might put "combatants" to shame
By swordless men whose coats are plain,
Who nobly duty's calls obey,
And grappling with disease and pain,
Face death and danger day by day.

Nor lack they aught the warrior claims,
The keen, brave eye, the fearless hand,
The purpose high, the glorious aims
(Things possible without command).
They pose not for applause or praise,
Yet not more high 'mong England's braves
Stands he who wields the sword that slays
Than he who guides the knife that saves.

### Christmas Competitions.

PARCELS have been received from Miss McEwen, Miss Mabel White, Maud Pound, Edith Price, Mabel Gilian, Mabel Eageas, Lily Baugh, Beatrice Stocks (these last six all scholars of the British School, Melton Mowbray), Miss G. Frems, Miss Edith Ford, Miss Ellen Wayte, Nurse S. E. Barker, Nurse Lowe (with such a kind letter we are going to pin it to the socks, for we are sure it will double their value as a gift), Nurse Baxter, Miss Edwards, Miss Roche, Nurse Barbara, Nurse H. Stork, Nurse Steer, Nurse Lee (a special gift for the Victor Ward at the London), Nurse Turner, Nurse Little, Nurse Firth, Miss Coates, Nurse M Reeve, Nurse Ayrton, Anna Lena, and C. G. and C. W., St. Albans. The awards will be announced next week. We could do with many more flannel petticoats and shirts if any of our readers could send us some to this office by Monday next. Warm clothing is such a precious gift to a patient just recovering from pneumonia or bronchitis, and going forth to work again.

### Presentation.

THERE was an interesting gathering at the Royal Free Hospital, Gray's Inn Road, on the 10th inst., when the nurses invited the members of the Committee and medical staff, with their lady friends, to afternoon tea, the occasion being to afford an opportunity for bidding adieu to Miss Eugénie Barton, the Lady Superintendent, who is leaving to be married to Captain Ludlow. Mr. Edward Ford North, the Chairman of the Weekly Board, in a few appropriate words, expressed the best wishes of his colleagues for Miss Barton's future happiness, and presented her with an engrossed and framed copy of a resolution passed by the Board, in which they expressed the high appreciation of the valuable services Miss Barton has rendered to the Royal Free Hospital during the past four years, and which have resulted in the high standard which at present characterises the nursing department of the institution. Miss Barton has been the recipient of many valuable presents, including a handsome travelling clock from members of the Committee, and a silver tea service from the nurses. Miss Henrietta Wedgwood, who has been for many years a Sister at King's College Hospital, and has been appointed Miss Barton's successor at the Royal Free Hospital, was also present on the occasion.

### Death in our Ranks.

ON the 11th inst., Nurse Lottie M'Lay, in faithful devotion to duty. Nurse M'Lay was a valued member of the Hillhead Nursing Institution, Glasgow.

### Notice.

WE are asked to state that from unforeseen circumstances, the Membership Examination of the Society of Operators in Massage and Medical Electricity is postponed. The date of the examination is not yet decided upon, but will be made known as soon as possible.

### Notes and Queries.

### Answers.

*The Nurses Bed.*—2s. received from Policy 294.

*Miss I.*—Guy's Hospital gives a certificate at the end of a year to probationers who pay a guinea a week, so does the Middlesex Hospital.

*Nurse Lily.*—Earlswood Asylum, or the Hants County Asylum, Knowle, Fareham. You must get a magistrate's order.

*Nurse Mary.*—Her Excellency Lady Harris, Government House, Bombay. We cannot answer queries privately, except under exceptional stress of circumstances.

### Wants and Workers.

*Acknowledgments.*—Several answers to the "wants" of "Alpha" and "Q" have been forwarded to them.

## Figures and Tables

**Fig. 1. f1:**
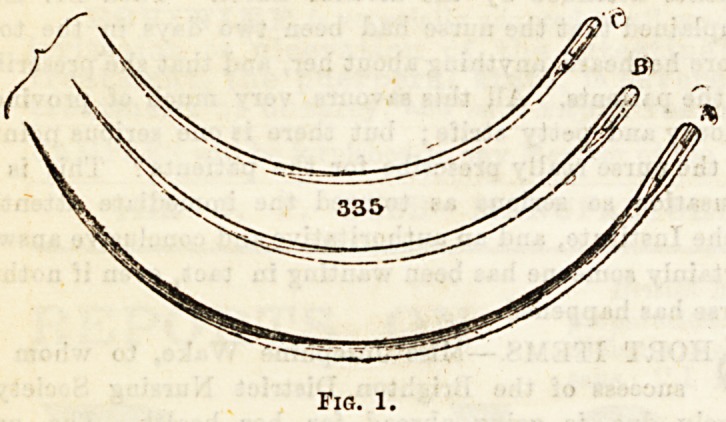


**Fig. 2. f2:**
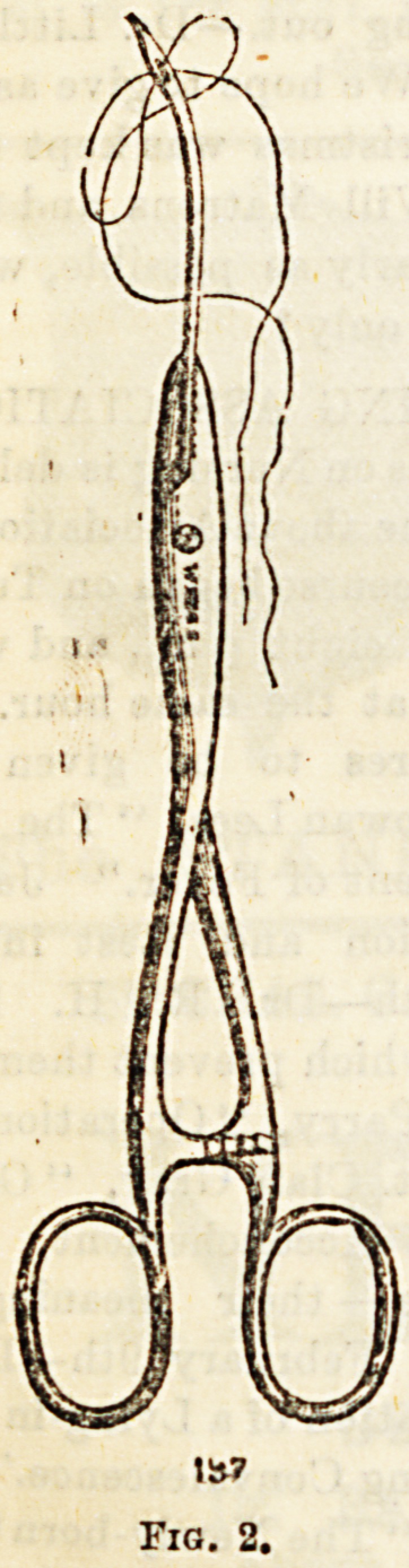


**Fig. 3. f3:**
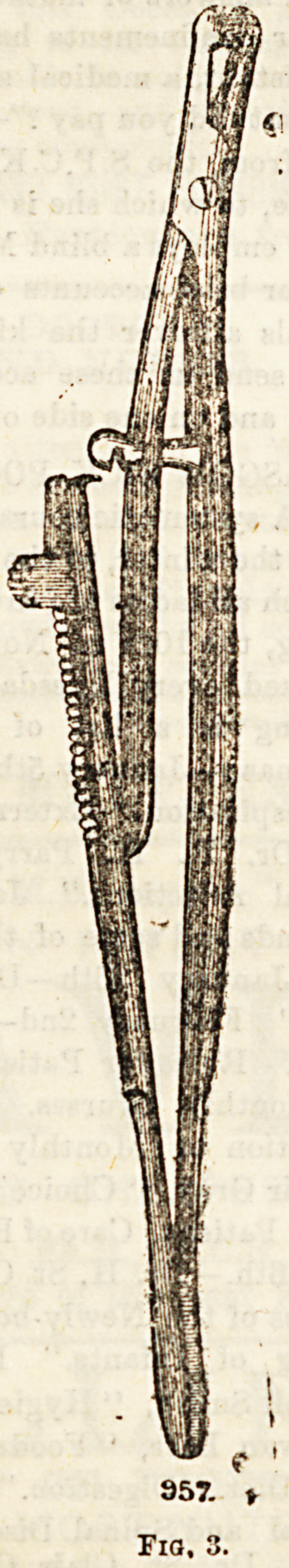


**Fig. 4. f4:**
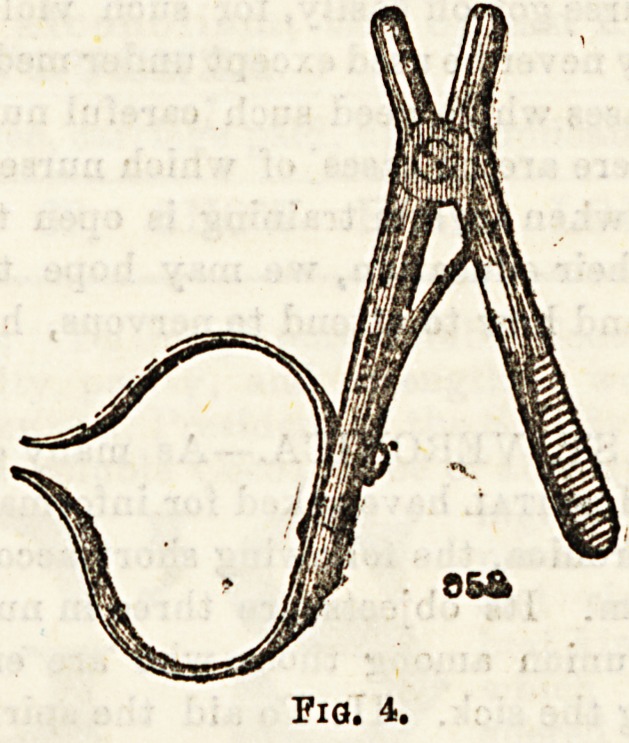


**Fig. 5. f5:**
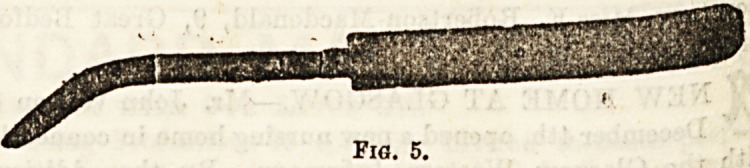


**Fig. 6. f6:**
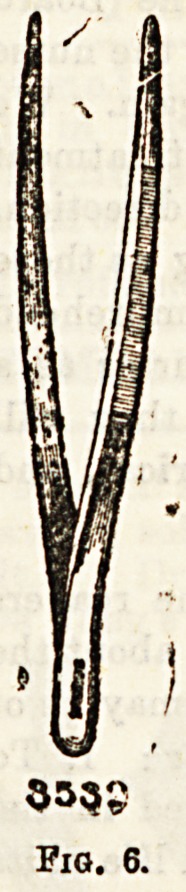


**Fig. 7. f7:**
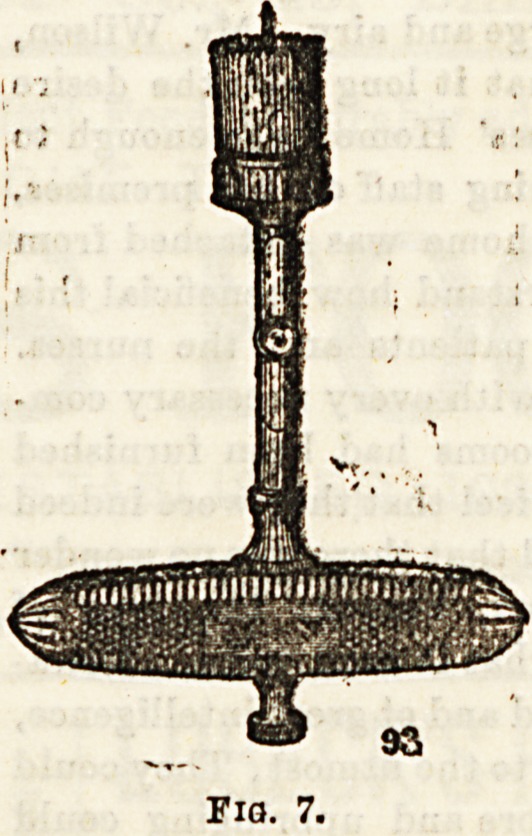


**Fig. 7a. f8:**
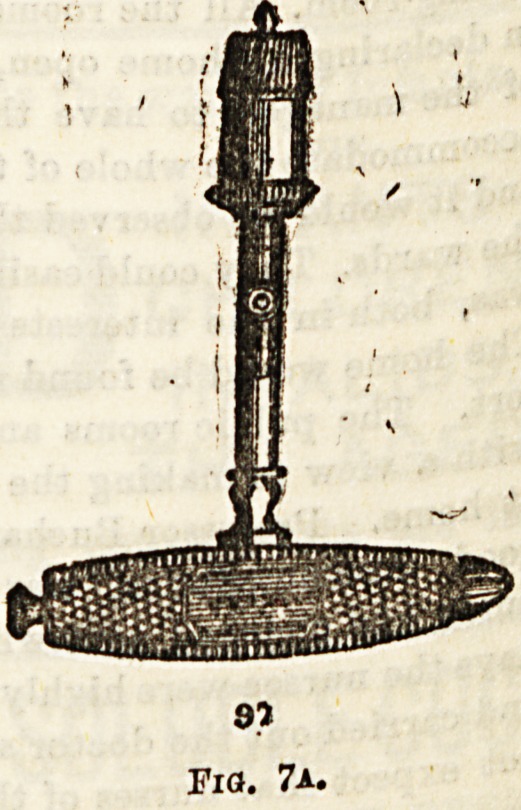


**Fig. 8. f9:**
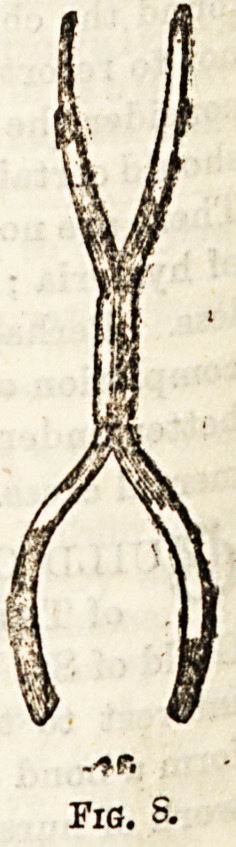


**Fig. 9. f10:**
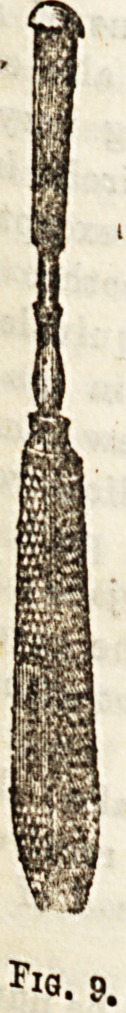


**Fig. 10. f11:**
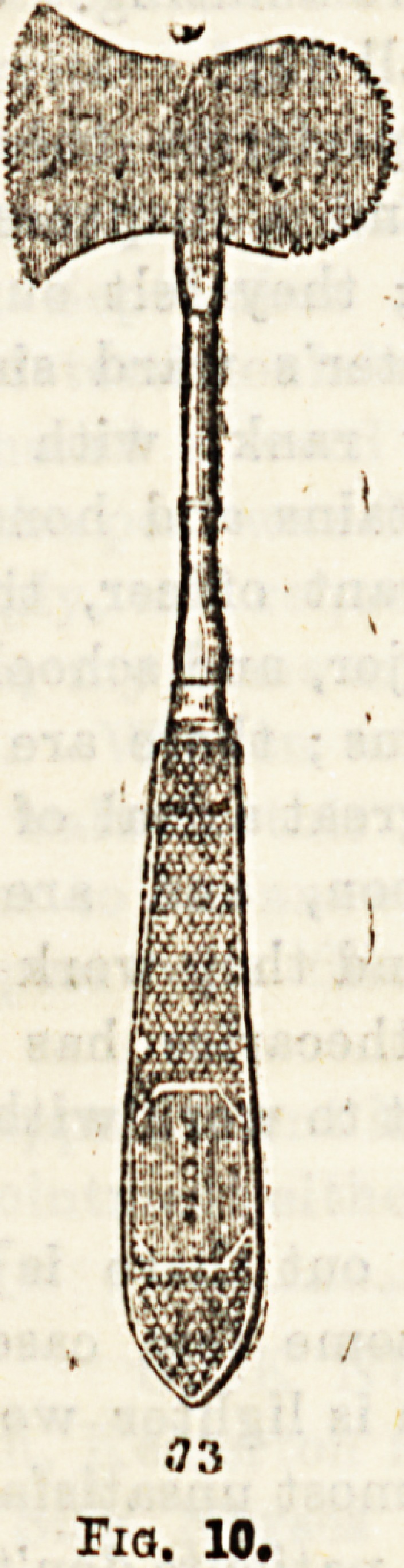


**Fig. 11. f12:**
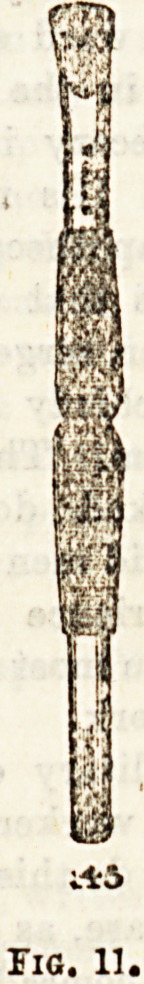


**Figure f13:**